# Supplementary material for: Circulating microRNAs as Promising Biomarkers in Colorectal Cancer
Source: Cancers (Basel). 2019 Jun 27;11(7):898. doi: 10.3390/cancers11070898 (PMC6679000; doi:10.3390/cancers11070898)
Supplement: Supplementary file 1 [file cancers-11-00898-s001.pdf]

Supplementary Materials: Circulating microRNAs as Promising Biomarkers in Colorectal Cancer

Óscar Rapado-González, Ana Álvarez-Castro, Rafael López-López, José Iglesias-Canle, María Mercedes Suárez-Cunqueiro and Laura Muinelo-Romay

Table S1. Main findings of the included studies about circulating miRNAs in patients with CRC.

Authors	Study Cohort	TNM Stage	Technique	Specimen	Molecular Profile	Sensitivity (%)	Specificity (%)	Expression Level	Normalization	Clinical Application
Ng et al. 2009 [1]	DP: 5 CRC, 5 HC VeP: 25 CRC, 20 HC VaP: 90 CRC, 50 HC, 20 IBD, 20 GC	I–IV	DP: Cancer MicroRNA Array with QuantiMir system VeP: SYBR green qRT-PCR VaP: SYBR green qRT-PCR	Plasma	miR-17-3p	64	70	▲	U6	Diagnosis
					miR-92a	89	70	▲		
Huang et al. 2010 [2]	DP: 20 CRC, 20 HC VaP: 100 CRC, 37 AA, 59 HC	I–IV	DP: qRT-PCR VaP: qRT-PCR	Plasma	miR-29a	69/62.2*	89.1/84.7*	▲	miR-16	Diagnosis
					miR-92a	84/64.9*	71.2/81.4*	▲		
Pu et al. 2010 [3]	103 CRC, 37 HC	I–IV	qRT-PCR	Plasma	miR-221	86	41	▲	No internal control	Diagnosis and prognosis
Cheng et al. 2011 [4]	DP: 74 CRC, 28 HC VaP: 111 CRC, 48 HC	I–IV	DP: qRT-PCR VaP: qRT-PCR	Plasma	miR-141	-	-	▲	cel-miR-39	Diagnosis and prognosis
Wang et al. 2012 [5]	DP: 10 CRC, 10 HC VaP: 90 CRC, 43 AA, 58 HC	I–IV	DP: miRCURY LNA™ Universal RT microRNA PCR system VaP: qRT-PCR	Plasma	miR- 601	69.2/72.1*	72.4/51.7*	▼	cel-miR-39	Diagnosis
					miR-760	80/69.8*	72.4/62.1*	▼		
Wang and Gu. 2012 [6]	36 non-metastatic CRC, 38 metastatic CRC	II–IV	qRT-PCR	Serum	miR-29a	75	75	▲	miR-16	Diagnosis
Giráldez et al. 2013 [7]	DP: 20 AA, 21 CRC, 20 HC VaP: 40 AA, 42 CRC, 53 HC	I–IV	DP: SAM-Bead Array platform VaP: qRT-PCR	Plasma	miR-19a, miR-29a, miR-15b, miR-335, miR-19b	-	-	▲	miR-16	Diagnosis
					miR-18a	72*	57*	▲		
Kanaan et al. 2013 [8]	DP: 20 CRC, 9 A, 12 HC VaP: 45 CRC, 16 A, 26 HC	I–IV	DP: 380 miRNA TaqMan low-density array card VaP: qRT-PCR	Plasma	miR-15b, miR-142-3p, miR-21, miR-532, miR-331, miR-652, miR-532-3p, miR-17, miR-195, miR-431, miR-139-3p	-	-	▲	U6	Diagnosis
Chen et al 2013 [9]	DP: 8 non-responder CRC, 8 responder CRC VaP: 36 non-responder CRC, 36 responder CRC	IV	DP: miRNA microarray VaP: qRT-PCR	Serum	miR-19a	66.7	63.9	▲	ath-miR-159	Predictive resistance to therapy
Toiyama et al. 2013 [10]	DP: 12 CRC, 12 HC VaP: 186 CRC, 43 A, 53 HC	I–IV	DP: qRT-PCR VaP: qRT-PCR	Serum	miR-21	91.9/81.13*	81.1/76.74*	▲	cel-miR-39	Diagnosis and prognosis
Liu et al. 2013 [11]	200 CRC, 50 AA, 80 HC	I–IV	qRT-PCR	Serum	miR-21	65	85	▲	miR-16	Diagnosis and prognosis
					miR-92a	65.5	82.5	▲		

Hofsli et al. 2013 [12]	DP: 30 CRC (IV), 10HC<br>VaP: 40 CRC (I-II), 10 HC	I–II<br>IV	DP: miRCURY LNA Universal RT miRNA PCR<br>VaP: qRT-PCR	Serum	miR-423-5p, miR-210, miR-720, miR-320a, miR-378, miR-92a, miR-29a	-	-	▲	Geometric mean	Diagnosis
					miR-155, miR-409-3p, miR-382, let-7d, miR-221, miR-425, miR-423-3p, miR-191, miR-107, miR-151-5p, miR-199a-3p, miR-103, miR-143, miR-106a	-	-	▼		
Brunet Vega et al. 2013 [13]	30 CRC, 26 HC	III	qRT-PCR	Serum	miR-18a, miR-29a	-	-	▲	miR-103, miR-16 and miR-let-7a	Diagnosis
Kjersem et al. 2014 [14]	DP: 12 responder CRC, 12 non-responder CRC<br>VaP: 90 responder CRC, 60 non-responder CRC	IV	DP: miRNA microarray<br>VaP: qRT-PCR	Plasma	miR-160a, miR-484, miR-130b	-	-	▲	miR-126, miR-15b and miR-24	Predictive resistance to therapy
Zhang et al. 2014 [15]	DP: 20 responder CRC, 20 non-responder CRC<br>TP: 20 responder CRC, 20 non-responder CRC<br>VaP: 93 responder CRC, 80 non-responder CRC		DP: TaqMan Array Human MicroRNA A+B Cards Set v3.0<br>TP: qRT-PCR<br>VaP: qRT-PCR	Serum	miR-20a, miR-130, miR-145, miR-216, miR-372	-	-	▲	serum volume	Predictive response to therapy
Yin et al. 2014 [16]	116 non-metastatic CRC, 108 metastatic CRC	I–IV	qRT-PCR	Serum	miR-126	77.78	68.97	▼	cel-miR-39	Diagnosis
					miR-141	86.11	76.11	▲		
					miR-21	73.61	66.38	▲		
Toiyama et al. 2014 [17]	DP: 24 CRC<br>VaP: 182 CRC, 24 HC	I–IV	DP: qRT-PCR<br>VaP: qRT-PCR	Serum	miR-200c	-	-	▲	cel-miR-39	Prognosis and tumor recurrence predictive
Lv et al. 2014 [18]	146 CRC, 60 HC	I–IV	qRT-PCR	Serum	miR-155	58.2	95	▲	miR-16	Diagnosis and prognosis
Ogata-Kawata 2014 [19]	DP: 88 CRC, 11 HC<br>VaP: 13 CRC, 8 HC	I–IV	DP: miRNA microarray<br>VaP: qRT-PCR	Serum	let-7a, miR-1229, miR-1246, miR-150, miR-21, miR-223, miR-23a	-	-	▲	miR-451	Diagnosis
Schou et al. 2014 [20]	138 CRC	IV	TaqMan Array Human MicroRNA cards A and B v2.0	Blood	miR-345	-	-	▲	Global mean normalization	Prognosis and predictive response to therapy
Yamada et al. 2015 [21]	DP: 24 CRN, 25 HC<br>VaP: 136 CRN, 52 HC	NA	DP: qRT-PCR<br>VaP: qRT-PCR	Serum	miR-21, miR-29a, miR-125b	-	-	▲	cel-miR-39	Diagnosis
Basati et al. 2015 [22]	55 CRC, 55 HC	I–IV	qRT-PCR	Serum	miR-194	72	80	▼	cel-miR-39	Diagnosis and prognosis
					miR-29b	77	75	▼		
Hur et al. 2015 [23]	169 CRC	I–IV	qRT-PCR	Serum	miR-885-5p	-	-	▲	cel-miR-39 and miR-16	Prognosis
Li et al. 2015 [24]	DP: 20 CRC, 20 HC<br>TP: 20 CRC, 20 HC<br>VaP: 65 CRC, 40 HC	II-III	DP: TaqMan Low Density Arrays<br>TP: qRT-PCR	Serum	miR-17-3p, miR-106a	-	-	▲	let-7d/g/i	Diagnosis and prognosis
					miR-145	-	-	▼		

	Ex-VaP: 90 CRC, 70 HC		VaP: qRT-PCR<br>Ex-VaP: qRT-PCR							
Yuan et al. 2015 [25]	118 CRC, 61 HC	I–IV	qRT-PCR	Plasma	miR-183	73.7	88.5	▲	U6	Diagnosis, prognosis and tumor recurrence predictive
Liu et al. 2015 [26]	98 CRC, 62 HC	I–IV	qRT-PCR	Serum	miR-592	-	-	▲	U6	Prognosis
Matsumura et al. 2015 [27]	DP: 6 CRC<br>VaP1: 90 CRC, 12 HC<br>VaP2: 209 CRC, 16 HC	I–IV	DP: miRNA microarray<br>VaP: qRT-PCR	Serum	miR-19a	-	-	▲	miR-16a	Prognosis and tumor recurrence predictive
Hansen et al. 2015 [28]	32 responder CRC, 31 non-responder CRC	IV	qRT-PCR	Plasma	miR-126-3p	-	-	▲	cel-miR-54 and cel-miR-238	Predictive resistance to therapy
Wang et al 2015 [29]	DP: 47 CRC, 33 HC<br>TP: 55 CRC, 57 HC<br>VaP: 22 CRC, 27 HC		DP: Human microRNA microarray<br>TP: qRT-PCR<br>VaP: qRT-PCR	Plasma	miR-7, miR-93, miR-25	-	-	▼	miR-1228	Diagnosis
					miR-409-3p	-	-	▲		
Kou et al. 2016 [30]	96 CRC, 48 HC	I–IV	qRT-PCR	Plasma	miR-23b	84.38	77.08	▼	U6	Diagnosis and prognosis
Liu et al. 2016 [31]	DP: 4 non-recurrent CRC, 3 recurrent CRC<br>VaP: 57 non-recurrent CRC, 27 recurrent	II–III	DP: RNA-seq<br>VaP: qRT-PCR	Serum	miR-4772-3p	-	-	▲	miR-16	Prognosis and tumor recurrence predictive
Vychitilova-Faltesjskova et al. 2016 [32]	DP: 144 CRC, 96 HC<br>TP: 80 CRC, 80 HC<br>VaP (diagnosis): 203 CRC, 100 HC<br>VaP (prognosis): 261 CRC	I–IV	DP: next generation sequencing (MiSeq Ilumina)<br>TP: qRT-PCR<br>VaP: qRT-PCR	Serum	miR-142-5p, miR-23a-3p, miR-376c-3p, miR-27a-3p	-	-	▲	miR-93-5p	Diagnosis and prognosis
Yu et al. 2016 [33]	DP: 3 responder CRC, 3 non-responder CRC<br>TP: 43 responder-CRC, 44 non-responder CRC<br>VaP: 26 responder CRC, 16 non-responder CRC	II–III	DP: miRNA microarray<br>TP: qRT-PCR<br>VaP: qRT-PCR	Serum	miR-345	-	-	▲	cel-miR-39	Predictive tumor response and prognosis
D'Angelo et al. 2016 [34]	16 responder CRC, 22 non-responder CRC	II–III	qRT-PCR	Serum	miR-125b	-	-	▲	miR-16 and cel-miR-39	Predictive tumor response
Zekri et al. 2016 [35]	TP: 30 CRC, 18 IBD, 18 polyps, 24 HC<br>VaP: 100 CRC, 24 HC		TP: Custom miScript miRNA PCR array<br>VaP: qRT-PCR	Serum	miR-223, miR-17, miR-19a, miR-19b, miR-20a	-	-	▲	SNORD 68	Diagnosis
Imaoka et al. 2016 [36]	DP: 3 CRC, 3 A, 3 HC<br>TP: 36 CRC, 12 A, 12 HC<br>VaP: 211 CRC, 56 A, 57 HC	I–IV	DP: miRNA expression microarray<br>TP: qRT-PCR	Serum	miR-1290	70.1/46.4*	91.2/91.2*	▲	cel-miR-39	Diagnosis, prognosis and

			VaP: qRT-PCR							tumor recurrence
Sun et al. 2016 [37]	DP: 40 CRC, 10 HC<br>VaP: 187 CRC, 47 HC	I–IV	DP: TaqMan® Array Human MicroRNA A + B Cards Set v3.0<br>VaP: qRT-PCR	Plasma	miR-141, let-7f-2, miR-628-5p, miR-96, miR-148a, miR-203, miR-200b, miR-22	-	-	▲	cel-miR-39 /miR-451	Diagnosis and prognosis
Ng et al. 2017 [38]	117 CRC, 90 HC	I–IV	qRT-PCR	Serum	miR-139-3p	96.6	97.8	▼	U6 and RNU48	Diagnosis
					miR-622	87.8	67.5	▲		
Tsukamoto et al. 2017 [39]	DP: 3 CRC, 3 HC<br>VaP: 326 CRC, 30 HC	I–IV	DP: miRNA microarrays<br>VaP: qRT-PCR	Serum	miR-21	-	-	▲	miR-16a	Diagnosis and prognosis
Zhu et al. 2017 [40]	54 non-metastatic CRC and 85 metastatic CRC	I–IV	qRT-PCR	Serum	miR-200b	77.78	68.97	▲	cel-miR-39	Diagnosis
					miR-141	86.11	76.11	▲		
					miR-200c	73.61	66.38	▲		
Pan et al. 2017 [41]	TP: 60 CRC, 60 HC<br>VaP: 80 CRC, 80 HC	I–IV	qRT-PCR	Plasma	miR-15b	50	68.75	▲	cel-miR-39 and miR-16-5p	Diagnosis
					miR-17	67.5	62.5	▲		
					miR-21	71.25	52.50	▲		
					miR-26b	72.50	56.25	▲		
					miR-145	62.50	61.25	▲		
Krawczyk et al. 2017 [42]	56 CRC, 70 HC	I–II	qRT-PCR	Plasma	miR-506	60.7	76.8	▲	U6	Diagnosis
					miR-4316	60.9	83.9	▲		
Wang et al. 2017 [43]	268 CRC, 102 HC	I–IV	qRT-PCR	Serum	miR-210	74.6	73.5	▲	miR-191-5p and U6	Diagnosis and prognosis
Ding et al. 2017 [44]	380 CRC, - HC	I–IV	qRT-PCR	Serum	miR-200, miR-141	-	-	▲	U6	Diagnosis
Wang et al. 2017 [45]	50 CRC, 50 HC	I–II	qRT-PCR	Plasma	miR-125a-3p, miR-320c	-	-	▲	miR-30e-5p	Diagnosis
Yan et al. 2017 [46]	DP: 3 CRC, 3HC<br>VaP: 77 CRC, 20 HC	I–IV	DP: miRNA microarrays<br>VaP: qRT-PCR	Serum	miR-638, miR-5787, miR-8075, miR-6869-5p, miR-548c-5p	-	-	▼	-	Diagnosis
					miR-486-5p, miR-3180-5p	-	-	▲		
Xu et al. 2017 [47]	103 CRC, 51 A, 100 HC	I–IV	qRT-PCR	Serum	miR-196b	87.38	63	▲<br>▼*	U6	Diagnosis and prognosis
Hur et al. 2017 [48]	DP: 186 CRC, 24 HC<br>VaP: 144 CRC, 24 HC	I–IV	DP: qRT-PCR<br>VaP: qRT-PCR	Serum	miR-203	-	-	▲	-	Prognosis
Maierthaler et al. 2017 [49]	DP: 20 non-metastatic CRC and 20 metastatic CRC<br>VaP: 309 non-metastatic CRC and 234 metastatic CRC	I–IV	DP: Custom TaqMan Array MicroRNA cards<br>VaP: qRT-PCR	Plasma	miR-200a, miR-200b, miR-122	-	-	▲	cel-miR-39	Prognosis
Tayel et al. 2018 [50]	30 CRC, 30 polyps, 30 HC	II–IV	qRT-PCR	Blood	miR-200c	86.67	73.33	▲	RNU48	Diagnosis and prognosis
Santasusagna et al. 2018 [51]	50 CRC	I–III	qRT-PCR	Plasma	miR-200a, miR-200b, miR-429	-	-	▲	miR-484	Prognosis
	TP: 40 CRC, 40 HC	I	qRT-PCR	Plasma	miR-182	78	91	▲	cel-miR-39	Diagnosis

Liu et al. 2018 [52]	VaP: 50 CRC, 50 HC, 50 A				miR-20a	-	-	▲	and U6	
Vafaee et al. 2018 [53]	DP: 75 CRC<br>VaP: 16 CRC, 8 HC		DP: OpenArray analysis<br>VaP: TaqMan Array Human MicroRNA Cards Set v2.0A/B	Plasma	let-7a, miR-21, miR-25, miR-30a	-	-	-	Quantile normalization	Prognosis
Yan et al. 2018 [54]	168 CRC, 20 HC	I–IV	miRNA microarray	Serum	miR-6803-5p	-	-	▲	miR-16	Diagnosis and prognosis
Liu et al. 2018 [55]	DP: 88 CRC, 11 HC<br>TP: 40 CRC, 40 HC<br>VaP: 80 CRC, 40 HC<br>Ex-VaP: 50 CRC, 50 A, 50 HC	I–IV	DP: qRT-PCR<br>TP: qRT-PCR<br>VaP: qRT-PCR<br>Ex-VaP: qRT-PCR	Plasma	miR-27a	80	77.5	▲	cel-miR-39	Diagnosis and prognosis
					miR-130a	70	80	▲		
Fu et al. 2018 [56]	18 CRC, 10 HC	I–IV	qRT-PCR	Serum	miR-17, miR-92a	-	-	▲	cel-miR-39	Diagnosis and prognosis
Wang et al. 2018 [57]	96 CRC, 60 HC	I–IV	qRT-PCR	Serum	miR-103	80	88.5	▲	U6	Diagnosis and prognosis
Ji et al. 2018 [58]	TP: 40 CRC<br>VaP: 226 CRC<br>Ex–VaP: 56 CRC	I–III	TP: miRNA Ready–to-Use PCR, Human Serum/Plasma Focus miRNA PCR Panel I and Panel II V1<br>VaP: qRT-PCR<br>Ex-VaP: qRT-PCR	Serum	miR-652-3p, miR-342-2p, miR-501-3p, miR-328-3p	-	-	-	miR-103-3p and miR-93-5p	Prognosis
Ulivi et al. 2018 [59]	52 CRC	I–IV	qRT-PCR	Plasma	miR-20b-5p, miR-29b-3p, miR-155-3p	-	-	▲	miR-484, miR-223-3p, cel-miR-39	Predictor tumor response and prognosis
Peng et al. 2019 [60]	108 CRC	I–IV	qRT-PCR	Serum	miR-548c-5p	-	-	▼	miR-16	Prognosis
Zhang et al. 2019 [61]	DP: 30 CRC pool, 10 HC pool<br>TP: 30 CRC, 30 HC<br>VaP: 79 CRC, 76 HC<br>Ex-VaP: 30 CRC, 30 HC	I–IV	DP: Exiqon miRCURY-Ready-to-Use PCR-Human-panel-I + II-V1.M<br>VaP: qRT-PCR<br>Ex-VaP: qRT-PCR	Plasma	miR-17-5p, miR-181a-5p, miR-18a-5p, miR-18b-5p	-	-	▲	cel-miR-39 and miR-16-5p	Diagnosis
Herreros-Villanueva et al. 2019 [62]	96 CRC, 101 AA, 100 HC	I–IV	qRT-PCR	Plasma	miR-19a, miR-19b, miR-15b, miR-29a, miR-335, miR-18a	-	-	▲	miR-1228 and cel-miR-39	Diagnosis
Sun et al. 2019 [63]	138 CRC, 60 HC	I–IV	qRT-PCR	Serum	miR-30a-5p	77.5	78.3	▼	cel-miR-39	Diagnosis and prognosis
Jin et al. 2019 [64]	25 non-responder CRC, 18 responder CRC	III–IV	qRT-PCR	Serum	miR-21-5p, miR-1246, miR-1229-5p, miR-96-5p	-	-	▲	miR-16	Predictive resistance to therapy

DP = discovery phase; TP = training phase; VeP = verification phase; VaP = validation phase; Ex-VaP = external-validation phase; qRT-PCR = quantitative real-time PCR; CRC = colorectal cancer; HC = healthy controls, IBD = inflammatory bowel disease; GC = gastric cancer; AA = advanced adenomas; A = adenomas. *Comparison AA/A vs HC.

Table S2. List of references of Figure 2.

miRNAs	References	miRNAs	References
miR-21	[10,11,19,21,39,41,65–80]	miR-720	[12,73]
miR-29 (a/b)	[2,6,7,12,13,21,22,62,76,77,81–84]	miR-191	[12,85]
miR-92 (a)	[1,2,11,12,68,76,77,82,84–87]	miR-409	[12,29]
miR-18 (a/b)	[7,13,61,62,70,76,85,88]	miR-155	[12,18]
miR-17	[1,24,35,41,61,82]	miR-378	[12,69]
miR-19 (a/b)	[7,24,35,62,79,87]	miR-199a	[12,78]
let-7 (a/d/f/g)	[12,19,68,72,89,90]	miR-423	[12,91]
miR-145	[24,41,72,81,84]	miR-1229	[19,80]
miR-106 (a/b)	[12,24,76,92,93]	miR-24	[85,91]
miR-20 (a)	[35,52,72,76,93]	miR-375	[86,94]
miR-223	[19,35,77,85,87]	miR-182	[52,95]
miR-23 (a/b)	[19,30,32,96]	miR-22	[70,89]
miR-125 (a/b)	[21,45,77,82]	miR-203	[68,89]
miR-103 (a)	[12,57,61,73]	miR-25	[29,70]
miR-320 (a/c/d)	[12,45,91,97]	miR-143	[12,76]
miR-200 (b/c)	[17,44,72,88,89]	miR-760	[5,86]
miR-141	[4,44,89,98]	miR-548 (c/at)	[46,99]
miR-221	[3,12,85]	miR-1246	[19,80]
miR-31	[68,72,98]	miR-34a	[12,100,101]
miR-139	[8,38,99]	miR-135 (a/b)	[72,102]
miR-15b	[7,41,62]	miR-26 (a/b)	[41,103]
miR-181 (a/b)	[61,68]	miR-142	[32,103]
miR-150	[19,100]	miR-335	[7,62]
miR-151 (a)	[12,61]	miR-1290	[36,97]
miR-107	[12,99]	miR-30 (a/b/c)	[63,104]
miR-210	[12,43]	miR-27a	[32,55]

Table S3. List of references of Figure 3.

miRNAs	References	miRNAs	References
miR-200 (a/b/c)	[17,37,49,51]	miR-210	[43]
miR-141	[4,37,51]	miR-203	[48]
miR-21	[10,39,53,105]	miR-372	[32]
miR-23 (a/b)	[30,32]	miR-155	[18]
miR-106a	[24,53]	miR-92a	[11]
let-7a	[53,106]	miR-122	[49]
miR-30a	[53,63]	miR-183	[25]
miR-548c	[60]	miR-221	[3]
miR-6803	[54]	miR-27a	[55]
miR-4772	[31]	miR-130a	[55]
miR-19a	[27]	miR-196b	[47]
miR-885	[23]	miR-185	[53]
miR-29b	[22]	miR-217	[53]
miR-194	[22]	miR-25	[53]
miR-96	[37]	miR-431	[53]
miR-17	[24]	miR-483	[53]
miR-592	[26]	miR-615	[53]
miR-26a	[107]	miR-892a	[53]
miR-124	[107]	miR-501	[58]
miR-103	[57]	miR-342	[58]
miR-376c	[32]	miR-652	[58]
miR-1290	[36]	miR-328	[58]

References

1. Ng, E.K.; Chong, W.W.; Jin, H.; Lam, E.K.; Shin, V.Y.; Yu, J.; Poon, T.C.; Ng, S.S.; Sung, J.J. Differential expression of microRNAs in plasma of patients with colorectal cancer: a potential marker for colorectal cancer screening. *Gut* **2009**, *58*, 1375–1381.
2. Huang, Z.; Huang, D.; Ni, S.; Peng, Z.; Sheng, W.; Du, X. Plasma microRNAs are promising novel biomarkers for early detection of colorectal cancer. *Int. J. Cancer* **2010**, *127*, 118–126.
3. Pu, X.; Huang, G.; Guo, H.; Guo, C.; Li, H.; Ye, S.; Ling, S.; Jiang, L.; Tian, Y.; Lin, T. Circulating miR-221 directly amplified from plasma is a potential diagnostic and prognostic marker of colorectal cancer and is correlated with p53 expression. *J. Gastroenterol. Hepatol.* **2010**, *25*, 1674–1680.
4. Cheng, H.; Zhang, L.; Cogdell, D.E.; Zheng, H.; Schetter, A.J.; Nykter, M.; Harris, C.C.; Chen, K.; Hamilton, S.R.; Zhang, W. Circulating plasma miR-141 is a novel biomarker for metastatic colon cancer and predicts poor prognosis. *PLoS One* **2011**, *6*, e17745.
5. Wang, Q.; Huang, Z.; Ni, S.; Xiao, X.; Xu, Q.; Wang, L.; Huang, D.; Tan, C.; Sheng, W.; Du, X. Plasma miR-601 and miR-760 are novel biomarkers for the early detection of colorectal cancer. *PLoS One* **2012**, *7*, e44398.
6. Wang, L.G.; Gu, J. Serum microRNA-29a is a promising novel marker for early detection of colorectal liver metastasis. *Cancer Epidemiol.* **2012**, *36*, e61-67.
7. Giráldez, M.D.; Lozano, J.J.; Ramírez, G.; Hijona, E.; Bujanda, L.; Castells, A.; Gironella, M. Circulating microRNAs as biomarkers of colorectal cancer: results from a genome-wide profiling and validation study. *Clin. Gastroenterol. Hepatol.* **2013**, *11*, 681–688.e3.
8. Kanaan, Z.; Roberts, H.; Eichenberger, M.R.; Billeter, A.; Ocheretner, G.; Pan, J.; Rai, S.N.; Jorden, J.; Williford, A.; Galandiuk, S. A plasma microRNA panel for detection of colorectal adenomas: a step toward more precise screening for colorectal cancer. *Ann. Surg.* **2013**, *258*, 400–408.
9. Chen, Q.; Xia, H.W.; Ge, X.J.; Zhang, Y.C.; Tang, Q.L.; Bi, F. Serum miR-19a predicts resistance to FOLFOX chemotherapy in advanced colorectal cancer cases. *Asian Pac. J. Cancer Prev.* **2013**, *14*, 7421–7426.
10. Toiyama, Y.; Takahashi, M.; Hur, K.; Nagasaka, T.; Tanaka, K.; Inoue, Y.; Kusunoki, M.; Boland, C.R.; Goel, A. Serum miR-21 as a diagnostic and prognostic biomarker in colorectal cancer. *J. Natl. Cancer Inst.* **2013**, *105*, 849–859.
11. Liu, G.H.; Zhou, Z.G.; Chen, R.; Wang, M.J.; Zhou, B.; Li, Y.; Sun, X.F. Serum miR-21 and miR-92a as biomarkers in the diagnosis and prognosis of colorectal cancer. *Tumour Biol.* **2013**, *34*, 2175–2181.
12. Hofsli, E.; Sjursen, W.; Prestvik, W.S.; Johansen, J.; Rye, M.; Tranø, G.; Wasmuth, H.H.; Hatlevoll, I.; Thommesen, L. Identification of serum microRNA profiles in colon cancer. *Br. J. Cancer* **2013**, *108*, 1712–1719.
13. Brunet Vega, A.; Pericay, C.; Moya, I.; Ferrer, A.; Dotor, E.; Pisa, A.; Casalots, À.; Serra-Aracil, X.; Oliva, J.C.; Ruiz, A.; et al. microRNA expression profile in stage III colorectal cancer: circulating miR-18a and miR-29a as promising biomarkers. *Oncol. Rep.* **2013**, *30*, 320–326.
14. Kjersem, J.B.; Ikdahl, T.; Lingjaerde, O.C.; Guren, T.; Tveit, K.M.; Kure, E.H. Plasma microRNAs predicting clinical outcome in metastatic colorectal cancer patients receiving first-line oxaliplatin-based treatment. *Mol. Oncol.* **2014**, *8*, 59–67.
15. Zhang, J.; Zhang, K.; Bi, M.; Jiao, X.; Zhang, D.; Dong, Q. Circulating microRNA expressions in colorectal cancer as predictors of response to chemotherapy. *Anticancer. Drugs* **2014**, *25*, 346–352.
16. Yin, J.; Bai, Z.; Song, J.; Yang, Y.; Wang, J.; Han, W.; Zhang, J.; Meng, H.; Ma, X.; Yang, Y.; et al. Differential expression of serum miR-126, miR-141 and miR-21 as novel biomarkers for early detection of liver metastasis in colorectal cancer. *Chin. J. Cancer Res.* **2014**, *26*, 95–103.
17. Toiyama, Y.; Hur, K.; Tanaka, K.; Inoue, Y.; Kusunoki, M.; Boland, C.R.; Goel, A. Serum miR-200c is a novel prognostic and metastasis-predictive biomarker in patients with colorectal cancer. *Ann. Surg.* **2014**, *259*, 735–743.
18. Lv, Z.; Fan, Y.; Chen, H.; Zhao, D. Investigation of microRNA-155 as a serum diagnostic and prognostic biomarker for colorectal cancer. *Tumor Biol.* **2015**, *36*, 1619–1625.
19. Ogata-Kawata, H.; Izumiya, M.; Kurioka, D.; Honma, Y.; Yamada, Y.; Furuta, K.; Gunji, T.; Ohta, H.; Okamoto, H.; Sonoda, H.; et al. Circulating exosomal microRNAs as biomarkers of colon cancer. *PLoS One* **2014**, *9*, e92921.
20. Schou, J. V.; Rossi, S.; Jensen, B. V.; Nielsen, D.L.; Pfeiffer, P.; Høgdall, E.; Yilmaz, M.; Tejpar, S.; Delorenzi, M.; Kruhøffer, M.; et al. miR-345 in metastatic colorectal cancer: a non-invasive biomarker for clinical

outcome in non-KRAS mutant patients treated with 3rd line cetuximab and irinotecan. *PLoS One* **2014**, *9*, e99886.
21. Yamada, A.; Horimatsu, T.; Okugawa, Y.; Nishida, N.; Honjo, H.; Ida, H.; Kou, T.; Kusaka, T.; Sasaki, Y.; Yagi, M.; et al. Serum miR-21, miR-29a, and miR-125b are promising biomarkers for the early detection of colorectal neoplasia. *Clin. Cancer Res.* **2015**, *21*, 4234–4242.
22. Basati, G.; Razavi, A.E.; Pakzad, I.; Malayeri, F.A. Circulating levels of the miRNAs, miR-194, and miR-29b, as clinically useful biomarkers for colorectal cancer. *Tumour Biol.* **2016**, *37*, 1781–1788.
23. Hur, K.; Toiyama, Y.; Schetter, A.J.; Okugawa, Y.; Harris, C.C.; Boland, C.R.; Goel, A. Identification of a metastasis-specific microRNA signature in human colorectal cancer. *J. Natl. Cancer Inst.* **2015**, *107*, doi:10.1093/jnci/dju492.
24. Li, J.; Liu, Y.; Wang, C.; Deng, T.; Liang, H.; Wang, Y.; Huang, D.; Fan, Q.; Wang, X.; Ning, T.; et al. Serum miRNA expression profile as a prognostic biomarker of stage II/III colorectal adenocarcinoma. *Sci. Rep.* **2015**, *5*, 12921.
25. Yuan, D.; Li, K.; Zhu, K.; Yan, R.; Dang, C. Plasma miR-183 predicts recurrence and prognosis in patients with colorectal cancer. *Cancer Biol. Ther.* **2015**, *16*, 268–275.
26. Liu, M.; Zhi, Q.; Wang, W.; Zhang, Q.; Fang, T.; Ma, Q. Up-regulation of miR-592 correlates with tumor progression and poor prognosis in patients with colorectal cancer. *Biomed. Pharmacother.* **2015**, *69*, 214–220.
27. Matsumura, T.; Sugimachi, K.; Iinuma, H.; Takahashi, Y.; Kurashige, J.; Sawada, G.; Ueda, M.; Uchi, R.; Ueo, H.; Takano, Y.; et al. Exosomal microRNA in serum is a novel biomarker of recurrence in human colorectal cancer. *Br. J. Cancer* **2015**, *113*, 275–281.
28. Hansen, T.F.; Carlsen, A.L.; Heegaard, N.H.H.; Sørensen, F.B.; Jakobsen, A. Changes in circulating microRNA-126 during treatment with chemotherapy and bevacizumab predicts treatment response in patients with metastatic colorectal cancer. *Br. J. Cancer* **2015**, *112*, 624–629.
29. Wang, S.; Xiang, J.; Li, Z.; Lu, S.; Hu, J.; Gao, X.; Yu, L.; Wang, L.; Wang, J.; Wu, Y.; et al. A plasma microRNA panel for early detection of colorectal cancer. *Int. J. Cancer* **2015**, *136*, 152–161.
30. Kou, C.H.; Zhou, T.; Han, X.L.; Zhuang, H.J.; Qian, H.X. Downregulation of miR-23b in plasma is associated with poor prognosis in patients with colorectal cancer. *Oncol. Lett.* **2016**, *12*, 4838–4844.
31. Liu, C.; Eng, C.; Shen, J.; Lu, Y.; Takata, Y.; Mehdizadeh, A.; Chang, G.J.; Rodriguez-Bigas, M.A.; Li, Y.; Chang, P.; et al. Serum exosomal miR-4772-3p is a predictor of tumor recurrence in stage II and III colon cancer. *Oncotarget* **2016**, *7*, 76250−76260.
32. Vychytilova-Faltejskova, P.; Radova, L.; Sachlova, M.; Kosarova, Z.; Slaba, K.; Fabian, P.; Grolich, T.; Prochazka, V.; Kala, Z.; Svoboda, M.; et al. Serum-based microRNA signatures in early diagnosis and prognosis prediction of colon cancer. *Carcinogenesis* **2016**, *37*, 941–950.
33. Yu, J.; Li, N.; Wang, X.; Ren, H.; Wang, W.; Wang, S.; Song, Y.; Liu, Y.; Li, Y.; Zhou, X.; et al. Circulating serum microRNA-345 correlates with unfavorable pathological response to preoperative chemoradiotherapy in locally advanced rectal cancer. *Oncotarget* **2016**, *7*, 64233−64243.
34. D'Angelo, E.; Fassan, M.; Maretto, I.; Pucciarelli, S.; Zanon, C.; Digito, M.; Rugge, M.; Nitti, D.; Agostini, M. Serum miR-125b is a non-invasive predictive biomarker of the pre-operative chemoradiotherapy responsiveness in patients with rectal adenocarcinoma. *Oncotarget* **2016**, *7*, 28647−28657.
35. Zekri, A.R.; Youssef, A.S.; Lotfy, M.M.; Gabr, R.; Ahmed, O.S.; Nassar, A.; Hussein, N.; Omran, D.; Medhat, E.; Eid, S.; et al. Circulating serum miRNAs as diagnostic markers for colorectal cancer. *PLoS One* **2016**, *11*, e0154130.
36. Imaoka, H.; Toiyama, Y.; Fujikawa, H.; Hiro, J.; Saigusa, S.; Tanaka, K.; Inoue, Y.; Mohri, Y.; Mori, T.; Kato, T.; et al. Circulating microRNA-1290 as a novel diagnostic and prognostic biomarker in human colorectal cancer. *Ann. Oncol.* **2016**, *27*, 1879–1886.
37. Sun, Y.; Liu, Y.; Cogdell, D.; Calin, G.A.; Sun, B.; Kopetz, S.; Hamilton, S.R.; Zhang, W. Examining plasma microRNA markers for colorectal cancer at different stages. *Oncotarget* **2016**, *7*, 11434–11449.
38. Ng, L.; Wan, T.M.; Man, J.H.; Chow, A.K.; Iyer, D.; Chen, G.; Yau, T.C.; Lo, O.S.; Foo, D.C.; Poon, J.T.; et al. Identification of serum miR-139-3p as a non-invasive biomarker for colorectal cancer. *Oncotarget* **2017**, *8*, 27393−27400.
39. Tsukamoto, M.; Iinuma, H.; Yagi, T.; Matsuda, K.; Hashiguchi, Y. Circulating exosomal microRNA-21 as a biomarker in each tumor stage of colorectal cancer. *Oncology* **2017**, *92*, 360–370.

40. Zhu, S.H.; He, X.C.; Wang, L. Correlation analysis of miR-200b, miR-200c, and miR-141 with liver metastases in colorectal cancer patients. *Eur. Rev. Med. Pharmacol. Sci.* **2017**, *21*, 2357–2363.
41. Pan, C.; Yan, X.; Li, H.; Huang, L.; Yin, M.; Yang, Y.; Gao, R.; Hong, L.; Ma, Y.; Shi, C.; et al. Systematic literature review and clinical validation of circulating microRNAs as diagnostic biomarkers for colorectal cancer. *Oncotarget* **2017**, *8*, 68317−68328.
42. Krawczyk, P.; Powrózek, T.; Olesiński, T.; Dmitruk, A.; Dziwota, J.; Kowalski, D.; Milanowski, J. Evaluation of miR-506 and miR-4316 expression in early and non-invasive diagnosis of colorectal cancer. *Int. J. Colorectal Dis.* **2017**, *32*, 1057–1060.
43. Wang, W.; Qu, A.; Liu, W.; Liu, Y.; Zheng, G.; Du, L.; Zhang, X.; Yang, Y.; Wang, C.; Chen, X. Circulating miR-210 as a diagnostic and prognostic biomarker for colorectal cancer. *Eur. J. Cancer Care (Engl).* **2017**, *26*, e12448.
44. Ding, M.; Zhang, T.; Li, S.; Zhang, Y.; Qiu, Y.; Zhang, B. Correlation analysis between liver metastasis and serum levels of miR-200 and miR-141 in patients with colorectal cancer. *Mol. Med. Rep.* **2017**, *16*, 7791–7795.
45. Wang, J.; Yan, F.; Zhao, Q.; Zhan, F.; Wang, R.; Wang, L.; Zhang, Y.; Huang, X. Circulating exosomal miR-125a-3p as a novel biomarker for early-stage colon cancer. *Sci. Rep.* **2017**, *7*, 4150.
46. Yan, S.; Han, B.; Gao, S.; Wang, X.; Wang, Z.; Wang, F.; Zhang, J.; Xu, D.; Sun, B. Exosome-encapsulated microRNAs as circulating biomarkers for colorectal cancer. *Oncotarget* **2017**, *8*, 60149–60158.
47. Xu, C.; Gu, L. The diagnostic effect of serum miR-196b as biomarker in colorectal cancer. *Biomed. Reports* **2017**, *6*, 39–45.
48. Hur, K.; Toiyama, Y.; Okugawa, Y.; Ide, S.; Imaoka, H.; Boland, C.R.; Goel, A. Circulating microRNA-203 predicts prognosis and metastasis in human colorectal cancer. *Gut* **2017**, *66*, 654–665.
49. Maierthaler, M.; Benner, A.; Hoffmeister, M.; Surowy, H.; Jansen, L.; Knebel, P.; Chang-Claude, J.; Brenner, H.; Burwinkel, B. Plasma miR-122 and miR-200 family are prognostic markers in colorectal cancer. *Int. J. Cancer* **2017**, *140*, 176–187.
50. Tayel, S.I.; Fouda, E.A.M.; Gohar, S.F.; Elshayeb, E.I.; El-sayed, E.H.; El-kousy, S.M. Potential role of microRNA 200c gene expression in assessment of colorectal cancer. *Arch. Biochem. Biophys.* **2018**, *647*, 41–46.
51. Santasusagna, S.; Moreno, I.; Navarro, A.; Martinez Rodenas, F.; Hernández, R.; Castellano, J.J.; Muñoz, C.; Monzo, M. Prognostic impact of miR-200 family members in plasma and exosomes from tumor-draining versus peripheral veins of colon cancer patients. *Oncology* **2018**, *95*, 309–318.
52. Liu, X.; Xu, T.; Hu, X.; Chen, X.; Zeng, K.; Sun, L.; Wang, S. Elevated circulating miR-182 acts as a diagnostic biomarker for early colorectal cancer. *Cancer Manag. Res.* **2018**, *10*, 857–865.
53. Vafaee, F.; Diakos, C.; Kirschner, M.B.; Reid, G.; Michael, M.Z.; Horvath, L.G.; Alinejad-Rokny, H.; Cheng, Z.J.; Kuncic, Z.; Clarke, S. A data-driven, knowledge-based approach to biomarker discovery: application to circulating microRNA markers of colorectal cancer prognosis. *NPJ Syst. Biol. Appl.* **2018**, *4*, 20.
54. Yan, S.; Jiang, Y.; Liang, C.; Cheng, M.; Jin, C.; Duan, Q.; Xu, D.; Yang, L.; Zhang, X.; Ren, B.; et al. Exosomal miR-6803-5p as potential diagnostic and prognostic marker in colorectal cancer. *J. Cell. Biochem.* **2018**, *119*, 4113–4119.
55. Liu, X.; Pan, B.; Sun, L.; Chen, X.; Zeng, K.; Hu, X.; Xu, T.; Xu, M.; Wang, S. Circulating exosomal miR-27a and miR-130a act as novel diagnostic and prognostic biomarkers of colorectal cancer. *Cancer Epidemiol. Biomarkers Prev.* **2018**, *27*, 746–754.
56. Fu, F.; Jiang, W.; Zhou, L.; Chen, Z. Circulating exosomal miR-17-5p and miR-92a-3p predict pathologic stage and grade of colorectal cancer. *Transl. Oncol.* **2018**, *11*, 221–232.
57. Wang, D.S.; Zhong, B.; Zhang, M.S.; Gao, Y. Upregulation of serum miR-103 predicts unfavorable prognosis in patients with colorectal cancer. *Eur. Rev. Med. Pharmacol. Sci.* **2018**, *22*, 4518–4523.
58. Ji, D.; Qiao, M.; Yao, Y.; Li, M.; Chen, H.; Dong, Q.; Jia, J.; Cui, X.; Li, Z.; Xia, J.; et al. Serum-based microRNA signature predicts relapse and therapeutic outcome of adjuvant chemotherapy in colorectal cancer patients. *EBioMedicine* **2018**, *35*, 189–197.
59. Ulivi, P.; Canale, M.; Passardi, A.; Marisi, G.; Valgiusti, M.; Frassineti, G.; Calistri, D.; Amadori, D.; Scarpi, E. Circulating plasma levels of miR-20b, miR-29b and miR-155 as predictors of bevacizumab efficacy in patients with metastatic colorectal cancer. *Int. J. Mol. Sci.* **2018**, *19*, 307.
60. Peng, Z.; Gu, R.; Yan, B. Downregulation of exosome-encapsulated miR-548c-5p is associated with poor prognosis in colorectal cancer. *J. Cell. Biochem.* **2019**, *120*, 1457–1463.

61. Zhang, H.; Zhu, M.; Shan, X.; Zhou, X.; Wang, T.; Zhang, J.; Tao, J.; Cheng, W.; Chen, G.; Li, J.; et al. A panel of seven-miRNA signature in plasma as potential biomarker for colorectal cancer diagnosis. *Gene* **2018**, *687*, 246–254.
62. Herreros-Villanueva, M.; Duran-Sanchon, S.; Martín, A.C.; Pérez-Palacios, R.; Vila-Navarro, E.; Marcuello, M.; Diaz-Centeno, M.; Cubiella, J.; Diez, M.S.; Bujanda, L.; et al. Plasma microRNA signature validation for early detection of colorectal cancer. *Clin. Transl. Gastroenterol.* **2019**, *10*, e00003.
63. Sun, Y.; Yang, B.; Lin, M.; Yu, H.; Chen, H.; Zhang, Z. Identification of serum miR-30a-5p as a diagnostic and prognostic biomarker in colorectal cancer. *Cancer Biomarkers* **2019**, *24*, 299–305.
64. Jin, G.; Liu, Y.; Zhang, J.; Bian, Z.; Yao, S.; Fei, B.; Zhou, L.; Yin, Y.; Huang, Z. A panel of serum exosomal microRNAs as predictive markers for chemoresistance in advanced colorectal cancer. *Cancer Chemother. Pharmacol.* **2019**, doi:10.3390/ijms19010307.
65. Wang, B.; Zhang, Q. The expression and clinical significance of circulating microRNA-21 in serum of five solid tumors. *J. Cancer Res. Clin. Oncol.* **2012**, *138*, 1659–1666.
66. Kanaan, Z.; Rai, S.N.; Eichenberger, M.R.; Roberts, H.; Keskey, B.; Pan, J.; Galandiuk, S. Plasma miR-21: a potential diagnostic marker of colorectal cancer. *Ann. Surg.* **2012**, *256*, 544–551.
67. Sazanov, A.A.; Kiselyova, E. V; Zakharenko, A.A.; Romanov, M.N.; Zaraysky, M.I. Plasma and saliva miR-21 expression in colorectal cancer patients. *J. Appl. Genet.* **2017**, *58*, 231–237.
68. Wang, J.; Huang, S.; Zhao, M.; Yang, M.; Zhong, J.; Gu, Y.; Peng, H.; Che, Y.; Huang, C. Identification of a circulating microRNA signature for colorectal cancer detection. *PLoS One* **2014**, *9*, e87451.
69. Zanutto, S.; Pizzamiglio, S.; Ghilotti, M.; Bertan, C.; Ravagnani, F.; Perrone, F.; Leo, E.; Pilotti, S.; Verderio, P.; Gariboldi, M.; et al. Circulating miR-378 in plasma: a reliable, haemolysis-independent biomarker for colorectal cancer. *Br. J. Cancer* **2014**, *110*, 1001–1007.
70. Wikberg, M.L.; Myte, R.; Palmqvist, R.; van Guelpen, B.; Ljuslinder, I. Plasma miRNA can detect colorectal cancer, but how early? *Cancer Med.* **2018**, *7*, 1697–1705.
71. Basati, G.; Emami Razavi, A.; Abdi, S.; Mirzaei, A. Elevated level of microRNA-21 in the serum of patients with colorectal cancer. *Med. Oncol.* **2014**, *31*, 205.
72. Eslamizadeh, S.; Heidari, M.; Agah, S.; Faghihloo, E.; Ghazi, H.; Mirzaei, A.; Akbari, A. The role of microRNA signature as diagnostic biomarkers in different clinical stages of colorectal cancer. *Cell J.* **2018**, *20*, 220–230.
73. Nonaka, R.; Miyake, Y.; Hata, T.; Kagawa, Y.; Kato, T.; Osawa, H.; Nishimura, J.; Ikenaga, M.; Murata, K.; Uemura, M.; et al. Circulating miR-103 and miR-720 as novel serum biomarkers for patients with colorectal cancer. *Int. J. Oncol.* **2015**, *47*, 1097–1102.
74. Bastaminejad, S.; Taherikalani, M.; Ghanbari, R.; Akbari, A.; Shabab, N.; Saidijam, M. Investigation of microRNA-21 expression levels in serum and stool as a potential non-invasive biomarker for diagnosis of colorectal cancer. *Iran. Biomed. J.* **2017**, *21*, 106–113.
75. Du, M.; Liu, S.; Gu, D.; Wang, Q.; Zhu, L.; Kang, M.; Shi, D.; Chu, H.; Tong, N.; Chen, J.; et al. Clinical potential role of circulating microRNAs in early diagnosis of colorectal cancer patients. *Carcinogenesis* **2014**, *35*, 2723–2730.
76. Luo, X.; Stock, C.; Burwinkel, B.; Brenner, H. Identification and evaluation of plasma microRNAs for early detection of colorectal cancer. *PLoS One* **2013**, *8*, e62880.
77. Liu, H.N.; Liu, T.T.; Wu, H.; Chen, Y.J.; Tseng, Y.J.; Yao, C.; Weng, S.Q.; Dong, L.; Shen, X.Z. Serum microRNA signatures and metabolomics have high diagnostic value in colorectal cancer using two novel methods. *Cancer Sci.* **2018**, *109*, 1185–1194.
78. Nonaka, R.; Nishimura, J.; Kagawa, Y.; Osawa, H.; Hasegawa, J.; Murata, K.; Okamura, S.; Ota, H.; Uemura, M.; Hata, T.; et al. Circulating miR-199a-3p as a novel serum biomarker for colorectal cancer. *Oncol. Rep.* **2014**, *32*, 2354–2358.
79. Zhu, M.; Huang, Z.; Zhu, D.; Zhou, X.; Shan, X.; Qi, L.; Wu, L.; Cheng, W.; Zhu, J.; Zhang, L.; et al. A panel of microRNA signature in serum for colorectal cancer diagnosis. *Oncotarget* **2017**, *8*, 17081–17091.
80. Guo, S.; Zhang, J.; Wang, B.; Zhang, B.; Wang, X.; Huang, L.; Liu, H.; Jia, B. A 5-serum miRNA panel for the early detection of colorectal cancer. *Onco. Targets. Ther.* **2018**, *11*, 2603–2614.
81. Ramzy, I.; Hasaballah, M.; Marzaban, R.; Shaker, O.; Soliman, Z.A. Evaluation of microRNAs-29a, 92a and 145 in colorectal carcinoma as candidate diagnostic markers: An Egyptian pilot study. *Clin. Res. Hepatol. Gastroenterol.* **2015**, *39*, 508–515.

82. Adams, S. V; Newcomb, P.A.; Burnett-Hartman, A.N.; Wurscher, M.A.; Mandelson, M.; Upton, M.P.; Zhu, L.C.; Potter, J.D.; Makar, K.W. Rare circulating microRNAs as biomarkers of colorectal neoplasia. *PLoS One* **2014**, *9*, e108668.
83. Li, L.; Guo, Y.; Chen, Y.; Wang, J.; Zhen, L.; Guo, X.; Liu, J.; Jing, C. The diagnostic efficacy and biological effects of microRNA-29b for colon cancer. *Technol. Cancer Res. Treat.* **2016**, *15*, 772–779.
84. Al-Sheikh, Y.A.; Ghneim, H.K.; Softa, K.I.; Al-Jobran, A.A.; Al-Obeed, O.; Mohamed, M.A.; Abdulla, M.; ABoul-Soud, M.A. Expression profiling of selected microRNA signatures in plasma and tissues of Saudi colorectal cancer patients by qPCR. *Oncol. Lett.* **2016**, *11*, 1406–1412.
85. Chang, P.Y.; Chen, C.C.; Chang, Y.S.; Tsai, W.S.; You, J.F.; Lin, G.P.; Chen, T.W.; Chen, J.S.; Chan, E.C. MicroRNA-223 and microRNA-92a in stool and plasma samples act as complementary biomarkers to increase colorectal cancer detection. *Oncotarget* **2016**, *7*, 10663–10675.
86. Elshafei, A.; Shaker, O.; Abd El-motaal, O.; Salman, T. The expression profiling of serum miR-92a, miR-375, and miR-760 in colorectal cancer: An Egyptian study. *Tumor Biol.* **2017**, *39*, 101042831770576.
87. Zheng, G.; Du, L.; Yang, X.; Zhang, X.; Wang, L.; Yang, Y.; Li, J.; Wang, C. Serum microRNA panel as biomarkers for early diagnosis of colorectal adenocarcinoma. *Br. J. Cancer* **2014**, *111*, 1985–1992.
88. Zhang, G.J.; Zhou, T.; Liu, Z.L.; Tian, H.P.; Xia, S.S. Plasma miR-200c and miR-18a as potential biomarkers for the detection of colorectal carcinoma. *Mol. Clin. Oncol.* **2013**, *1*, 379–384.
89. Sun Y, Liu Y, Cogdell D, Calin GA, Sun B, Kopetz S, Hamilton SR, Z.W. Examining plasma microRNA markers for colorectal cancer at different stages. *Oncotarget* **2016**, *1*, 379−384.
90. Ghanbari, R.; Mosakhani, N.; Sarhadi, V.K.; Armengol, G.; Nouraee, N.; Mohammadkhani, A.; Khorrami, S.; Arefian, E.; Paryan, M.; Malekzadeh, R.; et al. Simultaneous underexpression of let-7a-5p and let-7f-5p microRNAs in plasma and stool samples from early stage colorectal carcinoma. *Biomark. Cancer* **2015**, *7s1*, 39−48.
91. Fang, Z.; Tang, J.; Bai, Y.; Lin, H.; You, H.; Jin, H.; Lin, L.; You, P.; Li, J.; Dai, Z.; et al. Plasma levels of microRNA-24, microRNA-320a, and microRNA-423-5p are potential biomarkers for colorectal carcinoma. *J. Exp. Clin. Cancer Res.* **2015**, *34*, 86.
92. He, Y.; Wang, G.; Zhang, L.; Zhai, C.; Zhang, J.; Zhao, X.; Jiang, X.; Zhao, Z. Biological effects and clinical characteristics of microRNA-106a in human colorectal cancer. *Oncol. Lett.* **2017**, *14*, 830–836.
93. Chen, W.Y.; Zhao, X.J.; Yu, Z.F.; Hu, F.L.; Liu, Y.P.; Cui, B.B.; Dong, X.S.; Zhao, Y.S. The potential of plasma miRNAs for diagnosis and risk estimation of colorectal cancer. *Int. J. Clin. Exp. Pathol.* **2015**, *8*, 7092–7101.
94. Xu, L.; Li, M.; Wang, M.; Yan, D.; Feng, G.; An, G. The expression of microRNA-375 in plasma and tissue is matched in human colorectal cancer. *BMC Cancer* **2014**, *14*, 714.
95. Perilli, L.; Vicentini, C.; Agostini, M.; Pizzini, S.; Pizzi, M.; D'Angelo, E.; Bortoluzzi, S.; Mandruzzato, S.; Mammano, E.; Rugge, M.; et al. Circulating miR-182 is a biomarker of colorectal adenocarcinoma progression. *Oncotarget* **2014**, *5*, 6611–6619.
96. Karimi, N.; Ali Hosseinpour Feizi, M.; Safaralizadeh, R.; Hashemzadeh, S.; Baradaran, B.; Shokouhi, B.; Teimourian, S. Serum overexpression of miR-301a and miR-23a in patients with colorectal cancer. *J. Chinese Med. Assoc.* **2019**, *82*, 215–220.
97. Liu, X.; Xu, X.; Pan, B.; He, B.; Chen, X.; Zeng, K.; Xu, M.; Pan, Y.; Sun, H.; Xu, T.; et al. Circulating miR-1290 and miR-320d as novel diagnostic biomarkers of human colorectal cancer. *J. Cancer* **2019**, *10*, 43–50.
98. Wang, Y.; Chen, Z.; Chen, W. Novel circulating microRNAs expression profile in colon cancer: a pilot study. *Eur. J. Med. Res.* **2017**, *22*, 51.
99. Zhang, Y.; Li, M.; Ding, Y.; Fan, Z.; Zhang, J.; Zhang, H.; Jiang, B.; Zhu, Y. Serum microRNA profile in patients with colon adenomas or cancer. *BMC Med. Genomics* **2017**, *10*, 23.
100. Aherne, S.T.; Madden, S.F.; Hughes, D.J.; Pardini, B.; Naccarati, A.; Levy, M.; Vodicka, P.; Neary, P.; Dowling, P.; Clynes, M. Circulating miRNAs miR-34a and miR-150 associated with colorectal cancer progression. *BMC Cancer* **2015**, *15*, 329.
101. Nugent, M.; Miller, N.; Kerin, M.J. Circulating miR-34a levels are reduced in colorectal cancer. *J. Surg. Oncol.* **2012**, *106*, 947–952.
102. Wang, Q.; Zhang, H.; Shen, X.; Ju, S. Serum microRNA-135a-5p as an auxiliary diagnostic biomarker for colorectal cancer. *Ann. Clin. Biochem.* **2017**, *54*, 76–85.

103. Ghanbari, R.; Mosakhani, N.; Asadi, J.; Nouraee, N.; Mowla, S.J.; Yazdani, Y.; Mohamadkhani, A.; Poustchi, H.; Knuutila, S.; Malekzadeh, R. Downregulation of plasma miR-142-3p and miR-26a-5p in patients with colorectal carcinoma. *Iran. J. cancer Prev.* **2015**, *8*, e2329.
104. Ho, G.Y.F.; Jung, H.J.; Schoen, R.E.; Wang, T.; Lin, J.; Williams, Z.; Weissfeld, J.L.; Park, J.Y.; Loudig, O.; Suh, Y. Differential expression of circulating microRNAs according to severity of colorectal neoplasia. *Transl. Res.* **2015**, *166*, 225–232.
105. Menéndez, P.; Padilla, D.; Villarejo, P.; Palomino, T.; Nieto, P.; Menéndez, J.M.; Rodríguez-Montes, J.A. Prognostic implications of serum microRNA-21 in colorectal cancer. *J. Surg. Oncol.* **2013**, *108*, 369–373.
106. Liu, T.P.; Huang, C.C.; Yeh, K.T.; Ke, T.W.; Wei, P.L.; Yang, J.R.; Cheng, Y.W. Down-regulation of let-7a-5p predicts lymph node metastasis and prognosis in colorectal cancer: Implications for chemotherapy. *Surg. Oncol.* **2016**, *25*, 429–434.
107. Jinushi, T.; Shibayama, Y.; Kinoshita, I.; Oizumi, S.; Jinushi, M.; Aota, T.; Takahashi, T.; Horita, S.; Dosaka-Akita, H.; Iseki, K. Low expression levels of microRNA-124-5p correlated with poor prognosis in colorectal cancer via targeting of SMC4. *Cancer Med.* **2014**, *3*, 1544–1552.

© 2019 by the authors. Licensee MDPI, Basel, Switzerland. This article is an open access article distributed under the terms and conditions of the Creative Commons Attribution (CC BY) license (http://creativecommons.org/licenses/by/4.0/).
